# Genotype-based recall to study metabolic effects of genetic variation: a pilot study of *PPARG* Pro12Ala carriers

**DOI:** 10.1080/03009734.2017.1405127

**Published:** 2018-01-05

**Authors:** Prasad G. Kamble, Stefan Gustafsson, Maria J. Pereira, Per Lundkvist, Naomi Cook, Lars Lind, Paul W. Franks, Tove Fall, Jan W. Eriksson, Erik Ingelsson

**Affiliations:** a Department of Medical Sciences, Clinical Diabetes and Metabolism, Uppsala University, Sweden; b Department of Medical Sciences, Molecular Epidemiology and Science for Life Laboratory, Uppsala University, Sweden; c Department of Medical Sciences, Molecular Epidemiology, EpiHealth, Uppsala University, Sweden; d Department of Clinical Sciences, Genetic and Molecular Epidemiology Unit, Lund University Diabetes Centre, Malmö, Sweden; e Department of Medicine, Division of Cardiovascular Medicine, Stanford University School of Medicine, USA

**Keywords:** Genotype-based recall, metabolism, *PPARG* Pro12Ala

## Abstract

**Aim:**

To assess practical implications of genotype-based recall (GBR) studies, an increasingly popular approach for in-depth characterization of genotype–phenotype relationships.

**Methods:**

We genotyped 2500 participants from the Swedish EpiHealth cohort and considered loss-of-function and missense variants in genes with relation to cardiometabolic traits as the basis for our GBR study. Therefore, we focused on carriers and non-carriers of the *PPARG* Pro12Ala (rs1801282) variant, as it is a relatively common variant with a minor allele frequency (MAF) of 0.14. It has also been shown to affect ligand binding and transcription, and carriage of the minor allele (Ala12) is associated with a reduced risk of type 2 diabetes. We re-invited 39 Pro12Pro, 34 Pro12Ala, and 30 Ala12Ala carriers and performed detailed anthropometric and serological assessments.

**Results:**

The participation rates in the GBR study were 31%, 44%, and 40%, and accordingly we included 12, 15, and 13 individuals with Pro12Pro, Pro12Ala, and Ala12Ala variants, respectively. There were no differences in anthropometric or metabolic variables among the different genotype groups.

**Conclusions:**

Our report highlights that from a practical perspective, GBR can be used to study genotype–phenotype relationships. This approach can prove to be a valuable tool for follow-up findings from large-scale genetic discovery studies by undertaking detailed phenotyping procedures that might not be feasible in large studies. However, our study also illustrates the need for a larger pool of genotyped or sequenced individuals to allow for selection of rare variants with larger effects that can be examined in a GBR study of the present size.

## Introduction

The rapid increase in the prevalence of obesity and associated attributes, such as insulin resistance, is one of the major causes of the global epidemic of type 2 diabetes and related complications such as cardiovascular diseases (1). Genome-wide association studies (GWAS) have identified around 150 common gene variants related to obesity (2) and over 100 gene variants associated with type 2 diabetes (3), but their function remains largely elusive. Both genetic and environmental factors play an important role in the pathogenesis of metabolic diseases (4,5). Thus, it is imperative to understand whether the effect of a given variant of a genotype is conditional on context. The meticulous characterization of individuals carrying specific gene variants that have been implicated in the progression of insulin resistance and dyslipidemia can deliver knowledge of possible mechanisms which can further help in the diagnosis and management of metabolic diseases. In this perspective, genotype-based recall (GBR) studies can prove to be a powerful tool for comprehensive characterization of metabolic diseases (6).

The GBR method offers a participant recruitment approach for studies of genotype–phenotype relationships. The term refers to the prospective recruitment and phenotyping of subgroups of research participants with specific genotypes from a larger background cohort where the genotypes of all participants are known. The main advantage of the GBR approach is that a detailed phenotypic characterization can be done efficiently in a subgroup of participants, which may help maximize statistical power. The GBR design may be used both for assessment of genetic effects and gene–treatment interactions (6,7). For example, when treatment efficiency in randomized control trials is heterogeneous, or the treatment is expensive or could have deleterious side effects in a subgroup of patients, identification of underlying genetic components sometimes may not be possible in such trials as they are not always designed specifically for this purpose. In such cases, GBR could be a useful tool, as it is designed to test specific hypotheses about gene–treatment interaction. It can also provide information about underlying mechanisms and treatment efficacy between different genotype groups, which further can be used to optimize therapies.

This approach has been suggested by many investigators, but relatively few dedicated studies have been performed to date prospectively collecting data based on known genotypes. We are aware of a few studies in the cardiovascular and metabolic field, including a study of the relationship between the negative regulation of atrial natriuretic peptides by microRNA-425 (8), a study of functional effects of *ADIPOQ* gene variants (9), and a study by Tang and co-workers providing the proof of concept for the feasibility of individualized treatment using a GBR approach (10). A few previous studies of peroxisome proliferator-activated receptor gamma (*PPARG*) Pro12Ala (6,7,11–13), which is also a gene of interest in the present study, have employed a GBR-like approach.

In the present study, we aimed to test the feasibility and practical implications of applying a GBR approach to increase the understanding of a genetic variant associated with metabolic disease. We pursued this aim by re-inviting participants from an existing cohort study based on their carrier status of the *PPARG* Pro12Ala (rs1801282) variant to a smaller in-depth clinical investigation.

## Methods

### The EpiHealth study

EpiHealth is a population-based multicenter longitudinal cohort study, which has been conducted in the Uppsala and Malmö regions of Sweden. The primary goal of the EpiHealth cohort is to provide a resource to study interactions between several genotypes and lifestyle factors in a large cohort derived from the Swedish population within the age range of 45–75 years regarding development of common degenerative disorders, such as cardiovascular diseases, cancer, dementia, joint pain, obstructive lung disease, depression, and osteoporotic fractures. The EpiHealth cohort aims to collect data on 300,000 individuals.

To date, 14,000 individuals have been enrolled at the Uppsala test center. The design has been described in detail elsewhere (14); see also the study website (https://www.epihealth.se/) (15). Briefly, EpiHealth includes self-assessment of lifestyle factors using an internet-based questionnaire; a visit to a test center where blood samples are collected and physiological parameters are recorded; and follow-up of disease incidence via nationwide medical registers. The present study focused on participants enrolled at the EpiHealth test center in Uppsala before 7 March 2013, when the data extraction was performed for selection of DNA samples for genotyping.

### The ULSAM study

All men born between 1920 and 1924 in Uppsala, Sweden were invited to participate at age 50 in this longitudinal cohort study that was started in 1970. Participants were reinvestigated at the ages of 60, 71, 77, 82, and 88 years (16). Further details about ULSAM can be obtained online (http://www.pubcare.uu.se/ulsam) (17). Participants have undergone extensive phenotyping at repeated time points, including euglycemic clamps, oral glucose tolerance test (OGTT), DXA, echocardiography, 24 h ambulatory blood pressure measurement, and a range of biomarkers. For the present study, we used data on 922 non-diabetic participants who had been genotyped using genome-wide microarrays, and who underwent a euglycemic clamp at the age 71 examination. The total amount of glucose infused during a euglycemic clamp was taken as an index of sensitivity of subjects to an increasing concentration of plasma insulin. The glucose disposal rate (M) was then determined as the amount of glucose taken up during the last 60 min of the clamp. These data were used exclusively in the process of selecting a variant for the GBR study.

### Ethical approval

All participants in the EpiHealth and ULSAM studies provided written informed consents. A renewed consent was obtained from the EpiHealth participants for the present substudy, and all study protocols were approved by the Regional Ethics Review Board in Uppsala. A copy of informed consent is available on request.

### Genotyping

The 2500 most recently enrolled participants at the EpiHealth test center in Uppsala (before 7 March 2013) who had DNA available, with minimal adjustments of the sampling scheme to achieve an age and sex distribution reflecting the underlying distribution of EpiHealth, were selected for genotyping (Supplemental Table 1, available online) with the Illumina HumanCoreExome-12 v1.0 BeadChip including 522,731 autosomal markers. The genotype data were called using Illumina GenomeStudio 2011.1 GenCall followed by zCall version 3.3 (18). Sample exclusion filters applied prior to the genotype calling with zCall were: (1) discordant sex information when comparing reported sex and sex determined by the X-chromosome; (2) outlying, non-European ancestry based on the first two components in a multidimensional scaling analysis (>3 standard deviations [SD] from the mean); (3) outlying heterozygosity rate (>5 SD from the mean based on markers with a minor allele frequency [MAF] < 1% or markers with MAF ≥1%); and (4) low sample call rate (<98%). Markers with a call rate <97%, a Fisher’s exact test *p* value for Hardy–Weinberg equilibrium <10^−4^, a cluster separation score <0.4, or a GenTrain score <0.6 were also excluded. After genotype calling with zCall, markers with a call rate <99% or a Fisher’s exact test *p* value for Hardy–Weinberg equilibrium <10^−4^ were also excluded. In total, 2432 samples passed the quality control, and 2378 samples remained after further exclusion of related individuals. All quality filters were applied using PLINK v.1.0.7 (19). All participants in the ULSAM study provided blood samples for DNA analysis. DNA from the blood samples was extracted using a standard procedure, and genotyping was performed in the same fashion as described above.

### *The genotype-based recall study of* PPARG *Pro12Ala*

#### Participant recruitment for a GBR substudy

**Figure 1.**
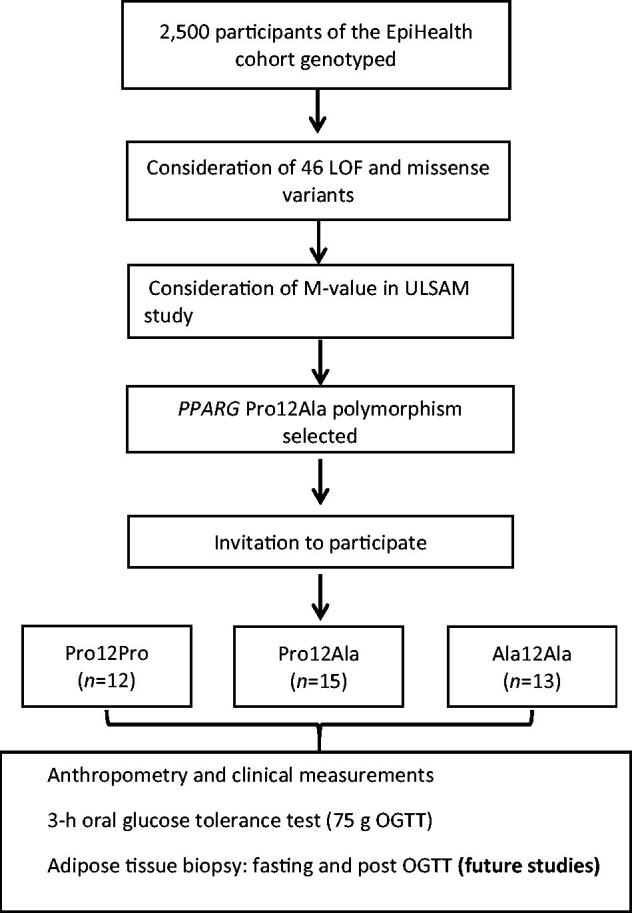
Schematic flow chart describing the study design.

We considered loss of function (LOF) and missense variants that were directly genotyped on the Illumina HumanCoreExome microarray as the possible basis for our GBR study and decided to study carriers and non-carriers of the *PPARG* Ala12 allele at rs1801282 (see Results for rationale for choosing this variant). Potential participants who reported being on treatment with an anticoagulant or lipid-lowering agent, or having diabetes, were excluded from further consideration as this might have convoluted the assessment of genotype–phenotype associations. For each participant with the Ala12Ala genotypes, age- and sex-matched carriers of the Pro12 allele (heterozygotes and homozygotes) were selected. Age matching was based on a 2-year interval around the birth date. Two batches of invitations were sent out. Participant recruitment was stopped after a second round of invitation because of the limited resource allocation to perform the in-depth clinical investigation for a larger number of participants in this feasibility study. Participants who agreed to participate were contacted by a nurse before they arrived at the diabetes outpatient clinic at the Uppsala University Hospital, Uppsala, Sweden, where a detailed clinical examination was done, and study-specific exclusion criteria were critically examined (Supplemental data, available online). All study participants, physicians, nurses, and researchers, except the database manager, involved in the study remained blinded to participant genotypes until all 40 individuals had completed the investigation. A schematic overview of the study design is shown in Figure 1.

#### Clinical examination

Participants arrived in the morning after overnight fasting and were examined by a study nurse and physician. Anthropometric measurements were done. A 3-h OGTT with 75 g of glucose was performed, and blood samples were collected at baseline, 15, 30, 60, 90, 120, and 180 min. Subcutaneous adipose tissue was obtained by needle biopsies at baseline and after the OGTT, to be used for future studies.

#### Anthropometric and biochemical measurements

Participants underwent a detailed investigation of their medical history and a physical examination, including height (cm), weight (kg), waist (cm) and hip (cm) circumference, which was carried out according to the World Health Organization guidelines. Body composition was determined by bioelectrical impedance (Kroppsanalysator BC-418MA, Tania). Body mass index (BMI) was calculated as weight (kg) divided by height (m^2^). A 3-h OGTT (75 g oral glucose load) was performed, and blood samples for analysis of glucose, insulin, non-esterified fatty acids (NEFA), and glycerol were drawn at baseline, 15, 30, 60, 90, 120, and 180 min. NEFA was measured using NEFA fluorometric assay kit (Cayman Chemicals, Anna Arbor, MI, USA), and glycerol was quantified using free glycerol reagent (Sigma Chemicals Co., St Louis, MO, USA). In addition, the fasting baseline blood sample was used for the analysis of lipid profile (triglyceride, total cholesterol, high-density cholesterol [HDL], and low-density lipoprotein [LDL]). Blood plasma and serum samples were analyzed at the Department of Clinical Chemistry, Uppsala University Hospital. Fasting glucose and insulin values were used to calculate a homeostatic model assessment of insulin resistance (HOMA-IR), which was used as an estimate of insulin resistance. In addition, glucose, insulin, and NEFA levels obtained during OGTT were used to calculate insulin sensitivity indices (ISI), which were determined by the Matsuda Index, and a revised quantitative insulin sensitivity check (QUICKI) (20–22). Plasma glucose at 2 h post-glucose load was used as an additional glucose assessment. The following formulas were used to calculate different insulin sensitivity indices. Revised QUICKI = 1/(log fasting glucose + log fasting insulin + log fasting NEFA); Matsuda Index = 10,000/√fasting glucose × fasting insulin × mean glucose × mean insulin.

#### Confirmatory genotyping

The fasting venous blood sample was collected in an EDTA tube and stored at −20 °C until DNA extraction using NucleoSpin Blood extraction kit (Macherey-Nagel GmbH & Co. KG, Düren, Germany). *De novo* genotyping of *PPARG* Pro12Ala polymorphism (rs1801282) was done to confirm the previous genotyping using single base primer extension with detection of the incorporated allele by fluorescent polarization template dye incorporation (23). Signal intensities were read using a Tecan Genios Pro fluorescence absorbance reader, and raw data from the fluorescence polarization were converted to genotype data using AlleleCaller 4.0.0.1.

### Statistical analysis

#### GBR variant selection

We used the ULSAM and EpiHealth cohorts for variant selection. Fasting plasma glucose and triglycerides were natural log transformed to promote normality. All metabolic phenotypes were *z*-score transformed (mean = 0 and SD =1) prior to the association tests to allow for comparisons of effect sizes across measures. Analyses were restricted to non-diabetic individuals (based on fasting plasma glucose and diabetes medication), and the association tests for all phenotypes measured in plasma were further restricted to individuals fasting for at least 6 h. We excluded first- or second-degree relatives and individuals of non-European ancestry from further analyses. The associations of each variant (independent variable) with each phenotype (dependent variable) were tested in linear regression models adjusting for age, sex (EpiHealth), and the first three (EpiHealth) or two (ULSAM) ancestry components (from multidimensional scaling analyses) assuming additive effects.

#### Analyses of clinical measures in the GBR study

In order to promote normal distribution, fasting glucose, fasting insulin, HOMA-IR, area under the curve (AUC) glucose, AUC insulin, triglycerides, AUC NEFA, AUC glycerol (0–120 min), and NEFA and glycerol at baseline were natural log transformed. Associations between a number of minor alleles and phenotypes were tested under an additive model using linear regressions adjusted for age, sex, and ancestry components. All statistical analyses were performed in STATA 13.1 (StataCorp LP, College Station, TX, USA) or R 3.0.0 (24).

## Results

### Selection of a gene variant as basis for the GBR study

In order to select the gene variant for our GBR study, we considered LOF and missense variants that were directly genotyped on the Illumina HumanCoreExome microarray. For this pilot study, we considered variants across the whole genome for which at least 10 participants were homozygous for the minor allele. According to these criteria, we identified 46 gene variants to be further considered (Table 1). We assessed associations of these 46 variants with clinical parameters in the EpiHealth cohort (*n* = 2500) and with M-values from clamp studies in ULSAM (*n* = 922) (Supplemental Table 2, available online). Among these variants, we opted to invite carriers and non-carriers of the *PPARG* Pro12Ala (rs1801282) to this pilot study, as it is a relatively common variant (MAF = 0.14) in the EpiHealth cohort, allowing inclusion of our projected number of 15 participants in each genotype group. Also, functionally, the Ala12 variant has a decreased transactivation capacity, and its association with cardiometabolic traits is well known. In addition, between the two significantly associated variants, Ala12 was strongly associated with the glucose disposal rate (M-value) as recorded in the euglycemic clamps of the ULSAM study (β = 0.19, *p* = 0.0032).

**Table 1.** Protein-altering variants in the EpiHealth cohort with ≥10 participants homozygous for minor alleles^a^.

rsID	Chromosome	Position (b37)	Minor/Major allele	N (aa)	N (aA)	Locus
rs1137101	1	66058513	A/G	531	1,090	*LEPR*
rs1935	10	64927823	G/C	528	1,113	*JMJD1C*
rs492594	2	169764176	C/G	474	1,038	*G6PC2*
rs13928	7	44153780	G/A	451	1,112	*AEBP1*
rs3784634	15	62259637	C/T	445	1,055	*VPS13C*
rs10851704	15	62202482	C/T	421	1,064	*VPS13C*
rs1208	8	18258316	G/A	377	1,074	*NAT2*
rs5215	11	17408630	C/T	367	1,034	*KCNJ11*
rs5219	11	17409572	T/C	366	1,035	*KCNJ11*
rs12529	10	5136651	G/C	303	1,014	*AKR1C3*
rs9938550	16	30999142	A/G	276	1,021	*HSD3B7*
rs1260326	2	27730940	T/C	261	980	*GCKR*
rs11057401	12	124427306	A/T	240	981	*CCDC92*
rs13266634	8	118184783	T/C	221	937	*SLC30A8*
rs1169288	12	121416650	C/A	216	1011	*HNF1A*
rs9814557	3	135720540	G/A	215	964	*PPP2R3A*
rs17197552	3	135722264	G/A	215	965	*PPP2R3A*
rs56200889	11	72408055	C/G	197	890	*ARAP1*
rs61748245	2	165476253	A/T	179	876	*GRB14*
rs2464196	12	121435427	A/G	177	935	*HNF1A*
rs1799930	8	18258103	A/G	172	931	*NAT2*
rs1137100	1	66036441	G/A	171	952	*LEPR*
rs6779903	3	135720851	T/G	153	847	*PPP2R3A*
rs321776	5	55407542	T/C	151	861	*ANKRD55*
rs17570	19	33878837	A/G	141	847	*PEPD*
rs1800437	19	46181392	C/G	104	779	*GIPR*
rs10761725	10	64974537	A/T	84	716	*JMJD1C*
rs17244632	2	165551404	A/G	75	670	*COBLL1*
rs17185413	11	61730553	C/T	71	675	*BEST1*
rs479661	2	169721377	G/A	65	590	*NOSTRIN*
rs7657817	4	89668859	T/C	60	615	*FAM13A*
rs9898682	17	41738823	A/G	52	445	*MEOX1*
rs12453522	17	41931375	G/A	49	555	*CD300LG*
rs10445686	2	135893372	G/A	45	477	*RAB3GAP1*
rs7607980	2	165551201	C/T	40	490	*COBLL1*
rs12440118	15	42744094	G/A	39	513	*ZFP106*
rs8940	7	116146074	G/C	35	514	*CAV2*
rs1801282	3	12393125	G/C	31	561	*PPARG*
rs7130656	11	45832509	G/A	30	477	*SLC35C1*
rs12702	15	44093927	C/T	24	315	*C15orf63*
rs328	8	19819724	G/C	17	372	*LPL*
rs12907567	15	62214607	T/C	13	284	*VPS13C*
rs11629598	15	62243197	C/T	13	284	*VPS13C*
rs2303405	15	62253791	C/T	13	281	*VPS13C*
rs10488698	11	116633947	A/G	13	285	*BUD13*
rs74459242	2	165578602	T/C	10	252	*COBLL1*

List of protein-altering variants annotated in the NHLBI Exome Sequencing Project.

a The genotype counts refers to unrelated individuals of European descent for which the association tests were performed.

### Participation rate in the GBR study

In total, we invited 39, 34, and 30 people with 0, 1, and 2 copies of the minor Ala12 allele, respectively. After sending two rounds of invitations, we included 12 Pro12Pro, 15 Pro12Ala, and 13 Ala12Ala participants with a response rate of 31%, 44%, and 40%, respectively (total *n* = 40), in this GBR study.

### *Clinical characteristics of carriers of* PPARG *Pro12Ala*

As should be expected from the modest sample size, we did not observe any significant differences in the anthropometric and clinical characteristics across the three different genotype groups (Table 2). Participants with the *PPARG* Pro12Ala variant were studied with an OGTT. The area under the curve for glucose, insulin, NEFA, and glycerol did not differ between the variant groups (Table 3). Likewise, the insulin sensitivity determined by calculating Matsuda Index, and revised QUICKI did not differ between the three groups of carriers (Table 3).

**Table 2.** Baseline characteristics of participants in the GBR study as genotyped by their carrier status of *PPARG* Pro12Ala^a^.

	GBR study			
Phenotype	Pro/Pro (*n* = 12)	Pro/Ala (*n* = 15)	Ala/Ala (*n* = 13)	*p* value
Age	64 (9)	63 (9)	64 (8)	0.92
Women (%)	8 (67)	9 (60)	9 (69)	0.92
BMI (kg/m^2^)	26.8 (3.3)	24.3 (3.2)	26.6 (3.6)	0.71
Waist–hip ratio	0.91 (0.06)	0.89 (0.08)	0.92 (0.07)	0.85
Fat percentage (%)	32.3 (8.8)	30.5 (6.6)	32.9 (7.1)	0.66
Fasting glucose (mmol/L)	5.88 (0.48)	5.83 (0.52)	5.89 (0.52)	0.57
Total cholesterol (mmol/L)	5.41 (0.60)	5.63 (0.80)	6.05 (0.90)	0.13
HDL cholesterol (mmol/L)	1.51 (0.34)	1.49 (0.31)	1.59 (0.27)	0.42
LDL cholesterol (mmol/L)	3.40 (0.54)	3.48 (0.65)	3.85 (0.93)	0.23
Triglycerides (mmol/L)	0.86 (0.27)	1.07 (0.48)	1.20 (0.48)	0.12

^a^The numbers given are either counts, percentages, or mean (standard deviation). The reported *p* values of the association between the phenotype and copies of the minor allele come from linear regressions adjusted for age, sex, and ancestry components under an additive model. The *p* value for age and sex comes from an ANOVA and a Fisher’s exact test, respectively. The beta from the regression is not shown. Use of medication is self-reported.

HDL: high-density lipoprotein; LDL: low-density lipoprotein. There was a varying number of missing observations for different phenotypes.

**Table 3.** Metabolic variables of participants in the GBR study as genotyped by their carrier status of *PPARG* Pro12Ala^a^.

Phenotype	Pro/Pro (*n* = 12)	Pro/Ala (*n* = 15)	Ala/Ala (*n* = 13)	*p* value
Fasting insulin (mU/L)	6.85 (4.06)	7.13 (3.36)	7.42 (3.20)	0.94
C-peptide (nmol/L)	0.68 (0.21)	0.70 (0.25)	0.67 (0.19)	0.33
HbA1c, IFCC (mmol/mol)	35.3 (3.0)	34.7 (3.2)	35.0 (3.6)	0.95
HOMA-IR	1.85 (1.33)	1.88 (0.98)	1.98 (0.98)	0.88
2h glucose (mmol/L)	8.61 (2.16)	7.63 (2.05)	8.95 (1.91)	0.75
Glucose, OGTT AUC_180 min × mmol/L_	1,503 (263)	1,335 (261)	1,493 (276)	0.82
Insulin, OGTT AUC_180 min × mU/L_	9,914 (7,311)	6,622 (3,269)	8,337 (5,680)	0.25
Matsuda index	5.35 (1.97)	6.66 (3.49)	5.64 (1.87)	0.43
Revised QUICKI	0.20 (0.01)	0.20 (0.01)	0.19 (0.01)	0.78
NEFA, OGTT AUC_120 min × µmol/L_	13,629.5 (7,073.83)	11,036.74 (3,810.39)	13,942.60 (5,467.38)	0.96
NEFA at baseline (µM)	206 (67)	218 (78)	243 (93)	0.57
Glycerol, OGTT AUC_120 min × µmol/L_	7,210.81 (2,967.45)	6,086.46 (2,977.29)	7,116.01 (2,967.64)	0.91
Glycerol at baseline (µM)	83.1 (28.7)	84.71 (46.0)	82.3 (33.6)	0.99

a The numbers given are either counts (percentages) or means (standard deviations). *p* values represent associations between number of minor alleles and phenotype under an additive model from linear regressions adjusted for age, sex, and ancestry components.

AUC: area under the curve; HOMA-IR: homeostatic model assessment of insulin resistance.

## Discussion

GWAS have made significant progress over the past decade in the discovery of genetic variants associated with disease risk (25). In the field of cardiovascular disease and type 2 diabetes research, hundreds of loci associated with these traits have been discovered. However, the functional impact of these gene variants on the phenotype needs more detailed follow-up studies using measurements that may not be feasible to undertake in large study populations where variants have been discovered. Here, we have performed a pilot study addressing the feasibility and practical implications of using a genotype-based recall approach to perform functional characterization of genetic variants established in GWAS. We genotyped 2500 individuals from the EpiHealth cohort, Uppsala, and identified 46 potential candidate variants based on *a priori* criteria. We decided to base our GBR study on the *PPARG* Pro12Ala polymorphism due to its relatively high minor allele frequency, its strong association with influenced glucose disposal rates in the ULSAM study, and its well-characterized role in the context of obesity, insulin resistance, and type 2 diabetes (26–29). As the genotypes of EpiHealth participants were already known, it was possible to invite a smaller number of individuals with the desired genotype distribution. This is an advantage of the GBR approach over conventional recruitment paradigms, where genotypes are randomly assigned, as GBR can be more powerful, particularly when rarer variants are of interest and when oversampling homozygotes of the rare variant. Another advantage of the GBR design is that you can match invitees on their baseline characteristics (in this case, age and sex) before inviting them to the substudy. This can be important to avoid confounding results, especially if factors other than the polymorphism itself are known to influence the phenotype. Similarly, in another GBR study of *PPARG* Pro12Ala by Stefan et al. (6), the participants were matched for age, sex, BMI, and waist–hip ratio, since the authors were evaluating the effect of free fatty acids (FFA) on insulin secretion and sensitivity and the above factors are known to influence these variables.

Besides its potential, the GBR approach also has considerable ethical challenges (30), mainly the potential disclosing of the genotype to the participants. For the present study, we considered ethical recommendations specific to GBR studies (31), in addition to the viewpoint of the local ethics committee at the Uppsala University. In brief, participants were informed about the reason and nature of this follow-up substudy in the invitation letter. However, although the participants were informed that they were being recruited to a genetic study, their Pro12Ala genotype was not disclosed to them. It would not have been easy to provide this information at the test center given that the staff was blinded to the genotype of the participant; but following the ethical principle of autonomy, if the participant had wanted to know their genotype, this would have been disclosed (by breaking the code). However, this did not occur in any case. We acknowledge that knowledge about the Pro12Ala variant being investigated in the present study may not be the most informative for a research participant, given the very small variance explained. Hence, the ethical considerations with regard to this specific study were very different to those of a study of more predictive variants, such as for example rare *BRCA* variants, where this information is of much higher importance. Thus the non-disclosure of carriership would be harder to justify, and the consequences of this knowledge for the participant and relatives would be much greater.

The participation rate is another important aspect of a GBR study, especially when studying rarer variants than Pro12Ala, and when the number of genotyped or sequenced individuals to recruit from is limited. As shown in our study, among the total number of participants genotyped (*n* = 2500), we had 561 Pro12Ala and 31 Ala12Ala carriers (Table 1). Based on our previous experience of a moderate participation rate (about 50%), which we almost reached, we realistically expected to recruit about 15 participants being homozygous for the Ala allele. This is also a reason for why we chose to recruit heterozygote carriers, even if the statistical power would be higher by contrasting only homozygotes. This highlights one of the challenges with GBR studies. Even for a relatively common variant as Pro12Ala (MAF = 14%) and a relatively large sampling frame (*n* = 2500), we did not have enough homozygotes to perform a study of only homozygotes. Indeed, among the uncommon and rare variants (MAF < 0.05) where the effects could be expected to be larger, we had five or fewer individuals homozygous for the risk allele (data not shown), making them unfeasible as basis for recruitment to a GBR. Hence, for future GBR efforts, a considerably larger cohort, at least 10-fold larger, and preferably sequenced to pick up all rare variants in the target gene, would be ideal.

*PPARG* is a nuclear transcription factor known to regulate adipogenesis and expression of genes involved in adipose tissue glucose and lipid metabolism (32). Two *PPARG* isoforms, *PPARG* 1 and *PPARG* 2, exist due to mRNA splicing. The difference between these two isoforms is that the latter isoform has an additional stretch of 28 amino acids towards its N-terminal (33). The expression of *PPARG* 1 is ubiquitous, whereas *PPARG* 2 is selectively expressed in adipose tissue (34). As a result of the amino acid substitution of proline to alanine, a common Pro12Ala polymorphism was identified at the 12th codon in exon B of *PPARG* (35). The frequency of the Ala variant varies from 4% to 28%, with higher prevalence among people of northern European ancestry (36). Previous studies have shown an association of the *PPARG* Pro12Ala polymorphism with improved insulin sensitivity and reduced risk of type 2 diabetes (26–28), but, in contrast, higher BMI (37,38). In addition to glucose metabolism, some studies have also indicated an association of the Pro12Ala polymorphism with lipid metabolism. Even if such associations have been inconsistent across different studies (39–44), there is evidence in our work in the Global Lipids Genetics Consortium (45) that there are associations of variation in *PPARG* with lipid fractions, presumably due to high correlations between insulin resistance and dyslipidemia. In line with our findings, a few prior studies characterizing the role of the *PPARG* Pro12Ala polymorphism using a GBR approach also failed to report any significant differences between the Pro12Pro and Pro12Ala participants for the phenotypes they studied, presumably due to a similarly low statistical power in these previous studies, as in ours. Specifically, a study by Stefan et al. (6) could not show any differences in insulin sensitivity in response to the FFA between the Pro12Pro and Pro12Ala groups, which they suggested could be due to the small number of participants included (10 Pro12Pro and 10 Pro12Ala). Another study by Pihlajamäki et al. (13) investigating the relation between the *PPARG* Pro12Ala and polyunsaturated fatty acids (PUFA) and its influence on serum lipid profile also employed the GBR approach. They were unable to find any effect of PUFA on the *PPARG* gene, which also remained unaffected by the diet–genotype interaction. Moreover, they could not find any differences in serum lipid profile and glucose between Pro12Pro and Ala12Ala individuals, and suggested that this was due to a small sample size. In line with these observations and as expected based on known effect sizes, we were unable to report any association of Pro12Ala polymorphism with measures of adiposity or insulin sensitivity.

In summary, we have performed a pilot study to test the feasibility and practical implications of applying a GBR approach to increase the understanding of cardiometabolic diseases. We did this by performing a comprehensive clinical study of individuals recruited based on their carriership for the *PPARG* Pro12Ala polymorphism. Due to the modest sample size, we observed no differences in anthropometric and metabolic parameters between the three groups of carriers. Our study suggests that the GBR approach is straightforward, and can be used as a tool to disentangle the nature of genotype–phenotype relationships. This approach can prove to be a valuable means to carry out a follow-up of studies where detailed, precise phenotyping in a large population is costly and time-consuming. Our study highlights several practical aspects of a GBR study, and that the approach is feasible. Specifically, no ethical concerns were raised by either the ethics committee or any participants. Further, the participation rate was unexpectedly high even though the study protocol was extensive in comparison to the less extensive prescreening in EpiHealth. Also, in spite of such an extensive study protocol, an in-depth examination was possible at a quite affordable cost in relation to examining all study individuals. Finally, the genotypes of all participants were confirmed in the recall phase. A different outcome of any of these four practical aspects could have falsified our hypothesis that GBR would be a workable strategy.

Our study also illustrates that a considerably larger number of genotyped—or preferably sequenced—individuals would be optimal as a basis for a more efficient GBR study. With a larger pool of genotyped samples, it would be possible to select more rare variants with larger effects, which would make the GBR more efficient and increase statistical power. Ultimately, it would be even better if the blood samples were subjected to DNA sequencing—either using whole-genome, exome, or targeted resequencing of the gene of interest—as that would also allow identification of carriers of rare or private mutations, which could be assumed to have even larger effects. Such rare or private mutations could then be grouped based on their inferred effects, for example, loss-of-function variant carriers could be studied in comparison with non-carriers. Given the limited number of samples (*n* = 2500) we had the resources to genotype in the present study, it was not feasible to consider rarer variants, and based on power calculations it was not surprising that we were not able to observe any significant effects of the chosen common variant with modest, or even minute, effects on the clinical phenotype.

## Supplementary Material

Supplemental data
