# Supplementary material for: Genotype-based recall to study metabolic effects of genetic variation: a pilot study of PPARG Pro12Ala carriers
Source: Ups J Med Sci. 2018 Jan 5;122(4):234–42. doi: 10.1080/03009734.2017.1405127 (PMC5810227; doi:10.1080/03009734.2017.1405127)
Supplement: Supplemental data [file IUPS_A_1405127_SM7034.docx]

**Supplemental Material**

**Genotype-based recall to study metabolic effects of genetic variation: A pilot study of PPARG Pro12Ala carriers**

Prasad G Kamble^1^, Stefan Gustafsson^2^, Maria J Pereira^1^, Per Lundkvist^1^, Naomi Cook^2^, Lars Lind^3^, Paul W Franks^4^, Tove Fall^2^, Jan W Eriksson^1*^ and Erik Ingelsson^2,5*^

^*^Contributed equally

**Supplemental text**

# Detailed inclusion/exclusion criteria

## Inclusion criteria

1. Males and females aged ≥ 18
2. The fasting plasma glucose should be in the range of 3-7.0 mmol/L on the morning of visit 1

### Exclusion criteria

1. Known diabetes with/without medication
2. Treatment with glucocorticoids or other immune modulating agents.
3. Acute myocardial infarction (NSTEMI, STEMI) or unstable angina, during the last 3 months
4. Stroke (ischemic and hemorrhagic) during the last 3 months
5. Cancer during the last 12 months
6. Other serious or acute illness as judged by the investigator, that may either put the subject at risk because of participation in the study, or influence the results or the subject’s ability to participate in the study
7. Pregnant or planning to be pregnant during the study
8. Known or suspected history of significant drug abuse
9. History of alcohol abuse or excessive intake of alcohol as judged by investigator
10. History of severe allergy/hypersensitivity or ongoing allergy/hypersensitivity, as judged by the investigator
11. Plasma donation within one month of screening or any blood donation during the 3 months prior to screening
12. Any other condition within the opinion of the investigator would render the patient unsuitable for inclusion in the study and /or for the patient’s safety
13. Involvement in the planning and/or conduct of the study
14. Judgment by the investigator that the subject should not participate in the study if considers subject unlikely to comply with study procedures, restrictions and requirements

**Supplemental Tables**

**Supplemental Table 1.** Baseline characteristics of the full EpiHealth cohort of participants enrolled at the Uppsala test center until the end of 2015, and the subset of participants selected for genotyping**^a^**

| **Phenotype*** | **Full EpiHealth cohort, Uppsala center (N=13,426)** | **Genotyped subset (N=2,500)** |
| --- | --- | --- |
| Age at blood draw (years) | 60.4 (8.5) | 61.2 (8.4) |
| Women (%) | 56.8 | 50.0 |
| BMI (kg/m^2^) | 26.2 (4.0) | 26.5 (3.9) |
| Waist-hip ratio | 0.89 (0.08) | 0.90 (0.08) |
| Body fat (%) | 30.7 (8.1) | 30.4 (8.2) |
| Plasma glucose (mmol/L) | 5.96 (1.01) | 6.00 (0.99) |
| Anti-diabetic medication (%) | 2.8 | 2.6 |
| Total cholesterol (mmol/L) | 5.87 (1.09) | 5.98 (1.11) |
| HDL cholesterol (mmol/L) | 1.55 (0.40) | 1.51 (0.39) |
| LDL cholesterol (mmol/L) | 3.81 (0.96) | 3.93 (0.98) |
| Triglycerides (mmol/L) | 1.22 (0.72) | 1.29 (0.75) |
| Lipid-lowering medication (%) | 10.2 | 10.2 |
| Systolic blood pressure (mmHg) | 133 (17) | 135 (17) |
| Diastolic blood pressure (mmHg) | 82 (9) | 83 (9) |
| Blood pressure-lowering medication (%) | 20.8 | 22.6 |

**^a^**The numbers given are either counts, percentages, or means (standard deviations). Use of medication is self-reported. A varying number of missing observations is present for different phenotypes. Abbreviations: HDL, high-density lipoprotein; LDL, low-density lipoprotein.

**Supplemental Table 2.** Associations between the protein-altering variants and measures of adiposity, glucose, and lipid metabolism in unrelated, non-diabetic individuals from the EpiHealth and ULSAM studies **^a^**
